# Emergence of natural and robust bipedal walking by learning from biologically plausible objectives

**DOI:** 10.1016/j.isci.2025.112203

**Published:** 2025-03-11

**Authors:** Pierre Schumacher, Thomas Geijtenbeek, Vittorio Caggiano, Vikash Kumar, Syn Schmitt, Georg Martius, Daniel F.B. Haeufle

**Affiliations:** 1 Max-Planck Institute for Intelligent Systems, Tübingen, Germany; 2 Hertie Institute for Clinical Brain Research and Center for Integrative Neuroscience, Tübingen, Germany; 3 Goatstream, Delft, the Netherlands; 4 Meta AI, New York, New York, USA; 5 Institute for Modelling and Simulation of Biomechanical Systems, University of Stuttgart, Stuttgart, Germany; 6 Department of Computer Science, University of Tübingen, Tübingen, Germany; 7 Institute of Computer Engineering, Heidelberg University, Heidelberg, Germany

**Keywords:** Behavioral neuroscience, Biological sciences, Neuroscience

## Abstract

Humans show unparalleled ability when maneuvering diverse terrains. While reinforcement learning (RL) has shown great promise for musculoskeletal simulation in the development of robust controllers, complex behaviors are only achievable under extensive use of motion data. We demonstrate that the combination of a recent RL algorithm with a biologically plausible reward is capable of learning controllers for 4 different musculoskeletal models and achieves locomotion with up to 90 muscles without demonstrations. Our controllers generalize to diverse and unseen terrains, while only a single adaptive objective function is needed for training. We validate our findings on four models in two different simulators. The RL agents perform robustly with complex 3D models, where reflex-controllers are difficult to apply, and produce close-to-natural motion. This is a first step for the motor control, biomechanics, and rehabilitation communities to generate complex human movements with RL, without using motion data or simple unrepresentative models.

## Highlights

• Biomechanical human models learn to walk close-to-naturally with RL and energy costs
• The learned controllers are robust under unseen conditions
• Our results hold for walking and running for 4 different models, with up to 90 muscles

## Graphical abstract

**Figure:**
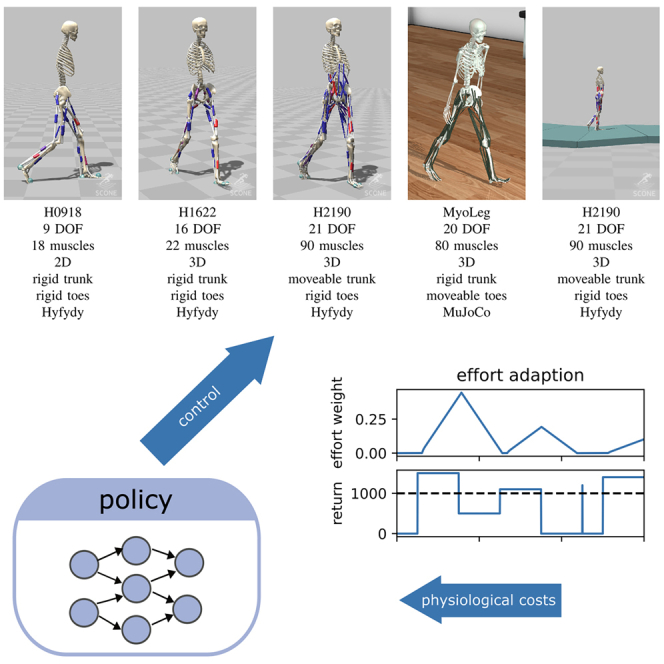

## Introduction

Humans excel at robust bipedal walking in complex natural environments by adequately adapting each movement and coordinating their muscles to compensate for uncertainties in ground conditions. In each step, they adequately tune the interaction of biomechanical muscle dynamics and neuronal signals to be robust against uncertainties in ground conditions.^1^ However, it is still not fully understood how the nervous system resolves the musculoskeletal redundancy to solve the multi-objective control problem considering stability, robustness, and energy efficiency. In computer simulations, energy minimization has been shown to be a successful optimization target, reproducing natural walking with trajectory optimization^2^ or reflex-based control methods.^3^^,^^4^^,^^5^^,^^6^ However, these methods focus on particular motions at a time and the resulting controllers are limited when compensating for perturbations.^6^^,^^7^^,^^8^^,^^9^ Trajectory optimization approaches using direct collocation also use energy as a cost term, however, they do not produce a feedback control strategy and are therefore limited in studying (neuronal) responses to an unexpected perturbation.^10^^,^^11^ In robotics, reinforcement learning (RL) methods recently achieved highly stable (and efficient) locomotion on quadruped systems^12^^,^^13^ and outperform optimal control approaches,^14^ but the generation of human-like walking with bipedal biomechanical models often relies on multi-camera marker-based motion capture data that is imitated by the controller.^15^^,^^16^^,^^17^ Some approaches are able to learn by imitation from a relatively small number of pre-recorded motions (<10)^16^^,^^17^^,^^18^ benefiting from faster convergence of RL algorithms with imitation components. In some cases, however, these controllers can be less robust.^19^ Additionally, the extension to behaviors unseen in the data is difficult with direct imitation learning and requires additional algorithmic complexity and large high-quality datasets.^20^^,^^21^ In a recent study^22^ behaviors were learned for a high-dimensional musculoskeletal model by imitating only 25 min of motion data, but it was not probed whether the generated motions and muscle activities represent human experimental data well. In one study^23^ human-like motions with RL are learned without explicitly tracking experimental trajectories, but require a complex curriculum schedule that might not transfer to other models.

Achieving natural locomotion with RL without sacrificing its robustness and generalization capability and while using realistic muscle activity patterns might pave the way for approaches that study human walking in complex natural environments.^24^

In this study, we demonstrate that biologically plausible objectives in combination with recent RL methods can directly (without learning by imitation) lead to the emergence of close-to-natural bipedal locomotion behaviors that can generalize to diverse terrains. In our work, we do not try to achieve human-like behavior with RL by attempting to follow recorded kinematic data^25^ but only by optimizing biologically plausible objectives in combination with realistic biomechanical constraints embedded into simulation engines. While we still use experimental data in this framework, the data are strongly aggregated into average gait cycles, and no individual trajectories are tracked. The use of biologically plausible objectives has a long history in motor control research.^26^ In contrast to direct trajectory tracking, metrics such as muscular effort, pain, and others are potentially more closely aligned with objectives that humans optimize during movement generation.^27^ The combination of a biomechanical system with these objectives under an RL paradigm has the potential to be general enough to allow for the reproduction of natural gait, similar to the achievements of reflex-based control,^4^^,^^28^^,^^29^ but with the potential for generating diverse and robust behaviors under many different conditions. Indeed, recent works^30^ have shown that RL algorithms may provide more robust controllers than model-predictive control approaches and might be able to handle the complexity of diverse movement generation with a high-dimensional physical embodiment.^20^^,^^22^

From a technical point-of-view, general reward terms have the benefit of being applicable to a broader range of behaviors without the need to collect specific trajectories. They might also enhance the controller’s reaction to perturbations, as objectives such as effort and pain minimization stay relevant even when deviating from the original motion. A simple trajectory tracking approach would be unable to compute rewards when the controller faces unseen situations.

The purpose of this study was to investigate if close-to-natural walking behaviors can be achieved with model-free RL using sufficiently good exploration^31^ combined with biologically plausible objectives (muscle effort, joint torques) and biomechanical human models. We investigated the quality of the produced gaits as well as the robustness of the controllers by comparing quantities derived from hip, knee and ankle joint angle trajectories as well as the GRFs of the feet. Experimental human data are used to modify the relative importance of the different metrics in the controller optimization, similar to.^32^^,^^33^^,^^34^ This goes beyond previous works applying RL to biomechanical models that either study low-dimensional systems,^23^ make use of human data^17^ during training, or learn unrealistic movements.^31^^,^^35^ The results are evaluated in four different models and two simulation engines of differing biomechanical complexity and accuracy using an identical training protocol and without changing the reward function.

## Results

RL agents were trained on four musculoskeletal models in 2D and 3D in order to achieve controllers that produce close-to-human motion and are robust against unseen variations. It was found that the obtained controllers performed better than comparable reflex-based baselines and were easily applied to highly complex models where no previous controllers were available. The highest value of the used gait quality metric was achieved by the RL controller for the H1622 model, which has 16 DOF and 22 muscles. The method achieved comparable results during walking with a speed of 1.2 m/s and running with a speed of 5.2 m/s.

### Reward function

Building on previous work on gait optimization,^5^ we found that a natural gait can be achieved with RL by using objectives that incentivize.

1. learning to maintain a given speed without falling down,
2. minimizing effort, and
3. minimizing pain (We do not use the term “pain” in the medical sense, but interpret it as an indication of mechanical loads potentially leading to injury).

Thus, our reward function contains three main terms:

(Equation 1) $$r=r_{\text{vel}}-c_{\text{effort}}-c_{\text{pain}}\text{,}$$

The first term specifies the external task the agent should solve. As we want the agent to move at a walking pace while keeping its balance, we chose the following objective:

(Equation 2) $$r_{\text{vel}}=\left\{ \begin{array}{l} \exp\left[-{\left(v-v_{\text{target}}\right)}^{2}\right]\;\text{if}\;v<v_{\text{target}}\\ 1\;\text{otherwise}, \end{array}\right.$$

where v is the center-of-mass velocity and the target velocity $v_{\text{target}}$ is chosen to be 1.2 ^m^/_s_, which is close to the average energetically optimal human walking speed.^36^ The velocity reward is constant above the target velocity to improve the optimization of the auxiliary cost terms, inspired by a recent study on reward shaping in robotics.^37^ While there is no reward gradient for the target speed above the threshold, we observed in our experiments that the strong effort costs used in training prevent the policy from reaching higher speeds, as doing so would require higher muscle activity.

Important for achieving natural human walking is the use of minimal muscle effort, as the literature suggests that energy efficiency is a key component of human locomotion:^38^^,^^39^

(Equation 3) $$c_{\text{effort}}=\alpha \left(t\right)\;a^{3}+w_{1}\;{\left(u-u_{\text{prev}}\right)}^{2}+w_{2}\;n_{\text{active}}$$

where the first term penalizes muscle activity a^40^ the second term incentivizes smoothness of muscle excitations u and the third term $n_{\text{active}}$ incentivizes a small number of active muscles (penalizing activity exceeding a certain value). The last term was included as experimental human data and prior work in reflex-based controllers has shown that the activity of muscle groups is small during the swing phase of steady-state gait and that there are low co-contraction levels of the leg muscles during the whole gait cycle.^28^ While an optimal solution to Equation 3 should use sparse muscle activity even without the last term, we found that it was necessary to add it in practice.

From a technical standpoint, it proved challenging to effectively minimize muscle activity. Using a strong cost scale causes a performance collapse in early training, even if that value would lead to energy-efficient walking in late training. We, therefore, chose an approach rooted in constrained optimization.^41^ We conjecture that a large initial action cost incentivizes near zero actions before any increase in the other reward terms is encountered, therefore preventing learning.

We propose an adaptation mechanism for the weighting parameter α(t), increasing the weight only when the agent performs well in the main task $\left(r_{\text{vel}}\right)$ and decreasing it when this constraint is violated. Concretely, we measure the performance by the task return. The details are provided in Algorithm 1, we marked the constrained optimization in blue.

Algorithm 1 **Effort weight adaptation**

**Require**: threshold θ, smoothing β, change in adaptation rate Δα, decay term λ∈[0,1]

 *r*_mean_← 0, $\alpha_{t}$ ← 0, *s*_mean_ ← 0

 **while** True **do**

 *r*← train episode()

 
$r_{\mathrm{mean}}\leftarrow \beta r_{\mathrm{mean}}+\left(1-\beta \right)r$

 
**if** $r_{\mathrm{mean}}>\theta$ **and** $s_{\mathrm{mean}}<0.5$ **then**

Δα←λΔα

 
**else if** *r*_mean_ >θ **and** *s*_mean_ >0.5 **then**

 
$\alpha_{t+1}\leftarrow \alpha_{t}+\Delta \alpha$

 
**else**

 
$\alpha_{t+1}\leftarrow \alpha_{t}+\Delta \alpha$

 
**end if**

 
$s_{\text{target}}\leftarrow \left\{ \begin{array}{c} 1\;\;if\;r_{\text{mean}}>\;\theta \\ 0\;otherwise \end{array}\right.$

 
$s_{\mathrm{mean}}\leftarrow \beta s_{\mathrm{mean}}+\left(1-\beta \right)s_{\mathrm{target}}$

 
**end while**

This adaptive learning mechanism is agnostic to the model and the task and removes the need for hand-tuning of schedules. A change in reward function over time could, however, destabilize learning, as previously collected environment transitions are not reflective of the current effort cost anymore.^42^ We, therefore, monitor the performance of the policy in the current environment, while the effort cost is only applied the moment when data are sampled from the replay buffer. This relabeling of previously collected data ensures that our off-policy algorithm can make efficient use of the full replay buffer.

The third term $c_{\text{pain}}$ is necessary to prevent unnatural optima. One striking example is the over-use of mechanical forces of the joint limits (e.g., massive knee over-extension) to keep a straight leg while minimizing muscle activity. As this is clearly unnatural behavior, we include objectives that account for the notion of pain:

(4) $$c_{\text{pain}}=w_{3}\;\sum_{i}\tau_{i}^{\lim}+w_{4}\;\sum_{j}F_{j}^{\text{GRF}}\text{,}$$

where $\tau_{i}^{\lim}$ is the torque with which the joint angle limit of joint i is violated (joint-limit pain) and $F_{j}^{\text{GRF}}$ is the vertical ground reaction force (GRF) for foot j (joint-loading pain). We only penalize GRFs if they exceed 1.2 times the model’s body weight^43^^,^^44^ such that all pain cost terms vanish close to the natural gait and do not further bias the solution. Joint limit violations in the simulation engines represent soft tissue in humans^45^ and are modeled by forces that push back against the violation.

We emphasize that the used cost weights are constant across all models and all joints and muscles. The weights $\omega_{i}$ for i∈{1,…,4} were found by first aligning the generated and experimental trajectories into gait cycles for each leg, starting and ending when the respective foot touches the ground. The result is then averaged over all gait cycles recorded from both legs. After this procedure, the data generated by the learned controllers is compared to its equivalent obtained from experimental human data.

The experimental match, defined as the fraction of the gait cycle for which the average simulated trajectory overlaps within the standard deviation of experimental data, serves as an optimization metric for our cost terms. The joint angles for the hips, knees and ankles of both legs are used to define this fraction. We note that the coefficients are identical across all joints and muscles, and stress that no human data were used **during** the learning process, but only to find weighting coefficients. This procedure is similar to Berret et al.^32^ with the difference that we search for one set of values that works across a range of models, instead of optimally for only one model.

Finally, we initialize the models with a randomized initial state that either starts with an elevated left or right leg and also contains a small amount of random noise, to mitigate asymmetries caused by the initial state distributions. We also clip all muscle excitations to lie between 0 and 0.5 at each time step, to further reduce muscle effort.

### Models

**Figure 1:**
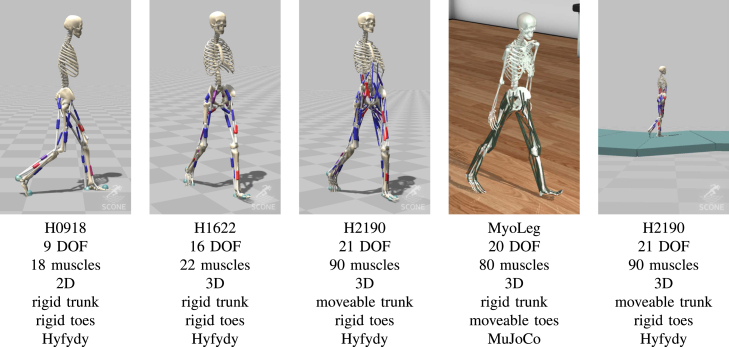
We achieve robust and energy-efficient natural walking with RL on a series of human models The different models differ in number of degrees of freedom (DOF), muscles, geometry, and simulation engine. We also use an uneven terrain environment. Videos: https://sites.google.com/view/naturalwalkingrl.

With the reward function and the RL approach described above, we are able to learn robust control policies for several models of human walking, with varying complexity, and across two different simulation engines with different levels of biomechanical accuracy (see Figure 1):

**H0918** A planar Hyfydy model with 9° of freedom (DOFs) and 18 muscles, based on Delp et al.^46^

**H1622** A 3D Hyfydy model with 16 DOFs and 22 muscles, based on Delp et al.^46^

**H2190** A 3D Hyfydy model with 21 DOFs and 90 muscles, and articulation between the otherwise rigid pelvis and torso, based on.^46^^,^^47^^,^^48^

**MyoLegV0** A 3D MuJoCo model with 20 DOFs and 80 muscles, based on Rajagopal et al.^47^ As for the *H0918* and *H1622* models, the pelvis and torso are one rigid body part. Additionally, each foot in the *MyoLegV0* contains articulated toes (all five toes are joined into one body segment). See Figure 1 for a summary of the models.

### Simulation engine

The simulation engines used for each model are indicated in the description and are either: (1) Hyfydy,^49^ which was used via the SCONE Python API^44^ or (2) MuJoCo,^50^ which was used via the MyoSuite^51^ environment. We chose these two engines, to highlight the versatility of our approach but also to bridge two communities: biomechanics and RL.

Hyfydy is an engine built for biomechanical accuracy. It is closely related to the well-established OpenSim^52^ framework, matching its level of detail in muscle and deformation-based contact-force models while providing increased computational performance. MuJoCo is a fast simulation framework widely used in the robotics and RL community. It offers a simplified muscle model with rigid tendons and resolves contact forces using the convex Gauss Principle. The MyoSuite^51^ builds on this framework, allowing for the development of high-dimensional muscle-control models which have recently gained a lot of interest from the RL community.^53^^,^^54^^,^^55^ Both engines achieve the required computational speed to train control policies for these high-dimensional models in under a day.

### Learned behaviors

**Figure 2:**
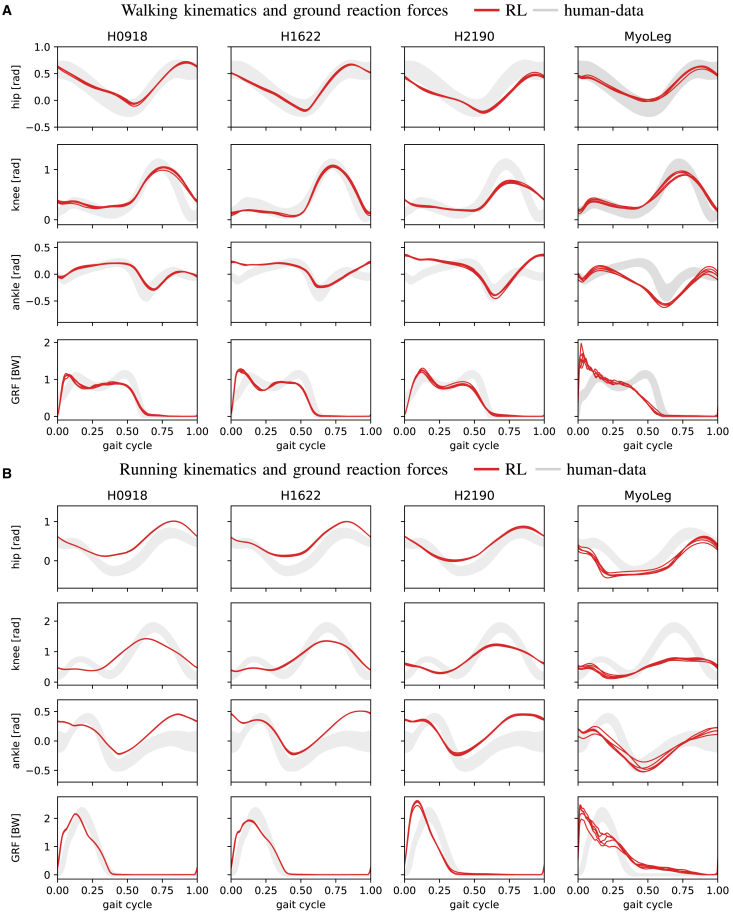
Gait-kinematics for RL agents for all models during walking and running Shown are the hip, knee, ankle and GRF values averaged over 5 rollouts of 10 s walking and running on flat ground. We excluded rollouts that did not achieve the whole episode length to clearly highlight the achieved kinematics. For walking, we observe slight discrepancies between experimental data (gray) and the RL behaviors (red), which are bigger for high-dimensional models. (A) The experimental data show human subjects walking at 1.2^m^/_s_ and is included in SCONE.^44^ (B) The experimental data show human subjects running at 5^m^/_s_ and was extracted from Hamner et al.^57^ For running, the behaviors deviate overall more from experimental data, a weak knee flexion can be observed. Nevertheless, the characteristic single-peaked GRF curve is present for all models, except for the *MyoLegV0*. The proposed reward function provides a strong and flexible starting point for researchers aiming to create robust and natural controllers for high-dimensional musculoskeletal systems. Also, see the videos on the website. In all simulations, the values (red) are averaged over both legs and 5 rollouts of 10 s are recorded. The experimental data (shaded) is shown as maximum and minimum values over all subjects.

We first show that with our framework, we can train agents across 4 different models to produce walking gaits with the same training approach and reward function. In Figure 2 we compare the resulting gait kinematics against experimental data.^56^ Kinematics are shown for 5 rollouts of the most human-like policy checkpoint that was achieved over the entire training run over 10 random seeds, averaged over all gait cycles of both legs in a 10 s rollout (For videos, see: https://sites.google.com/view/naturalwalkingrl). The gait data for locomotion contains participants walking at 1.2 m/s and participants running at 5.2 m/s.

**Figure 3:**
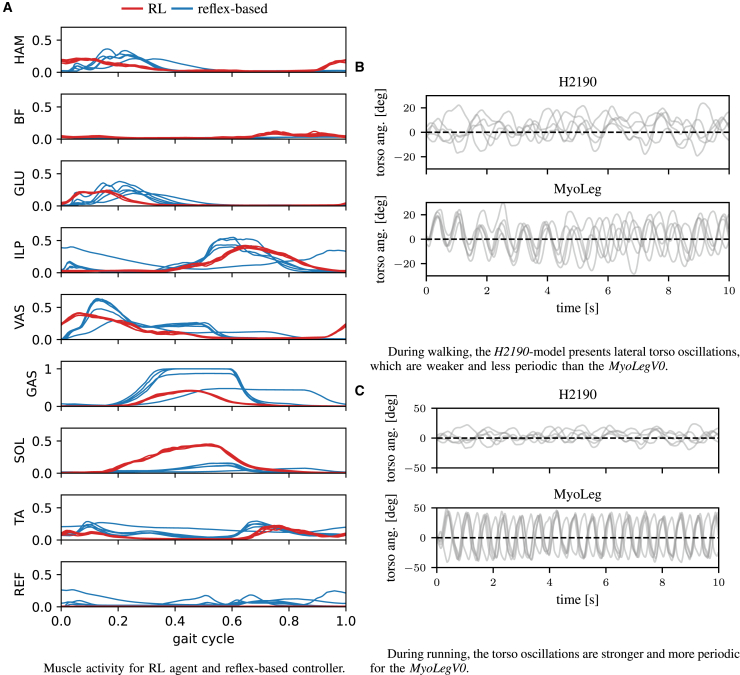
Muscle activity and torso oscillations for the RL agents We compare muscle activities for two controller types for natural walking with the *H0918* model. (A) The activity for the RL agent has been clipped to 0.5. We use 5 roll-outs of the most natural RL policy and 5 reflex-based controllers that were optimized until convergence. The initial state for the RL agent is randomized, which would cause collapse with the reflex controllers, as they are sensitive to the initial state. (B) and (C) We show the torso angle with the vertical axis for 5 rollouts of 10 s for the *H2190*and the *MyoLegV0* models for walking and for running. The *MyoLegV0* presents stronger lateral oscillations. The dashed line shows a straight torso posture.

The results for the planar *H0918* and the 3D *H1622* model look very similar to the experimental data. While the agents achieve the most human-like gaits here, the models are also of limited complexity and applicability, compared to the high-dimensional systems, *H2190* and *MyoLegV0*. As seen in Table 1 and Figure 2 our approach still achieves periodic gaits resembling human kinematics with the difficult-to-control 80 and 90 muscle models, even though they contain more artifacts. The *H2190*-agent exhibits less knee flexion and the *MyoLegV0*-agent lacks the double-peaked GRF structure; it produces periodic torso oscillations, see Figure 3. Overall, the behavior of the *H2190* model appears more natural than the one produced with the *MyoLegV0* model, see also the discussion and the supplementary videos. Both models produce agents with ankle profiles deviating slightly from experimental data.

**Table 1:** Average cubic muscle activity (effort), the percentage match with human experimental data (exp. match), and the average distance walked on the uneven terrain (Figure 1)

controller	system	avg. effort	experimental match	avg. distance [m]
reflex	H0918	$0.041\pm 3\times 10^{-3}$	0.68±0.08	2.46±0.98
RL	H0918	$0.013\pm 3\times 10^{-4}$	0.67±0.03	10.42±0.94
RL	H1622	$0.015\pm 2\times 10^{-4}$	0.73±0.01	5.6±0.99
RL	H2190	$0.017\pm 2\times 10^{-5}$	0.50±0.01	10.59±2.51
RL	MyoLeg	$0.013\pm 2\times 10^{-4}$	0.43±0.05	n.a

Note that the exp. match metric measures the percentage of the gait cycle during which the trajectory perfectly lies inside the standard deviation of the experimental data. Even relatively natural gaits can still achieve a low metric if the angles are slightly shifted. Reported are mean ± SD over 5 rollouts of 10 s with the best policy.

Nevertheless, Table 1 shows that RL gaits not only approximate human walking but are also robust and energy-efficient across all models, without changes in the reward function and only minimal changes in the hyperparameters of the RL method. We provide the training curves and additional metrics for 10 random seeds in Figure S1.

In order to probe the robustness of our controllers, we perform rollouts on uneven terrain, which was **not** seen during training. The entire training procedure was performed with flat ground. The generated terrain contains 10 tiles of 1 m length with random slopes of ±5° and is fixed for all evaluations. The behavior of the planar *H0918*-model is compared against a popular reflex-based controller as an illustrative example, adapted from^28^^,^^44^ and included with the SCONE software. We were only able to use this simple reflex-based controller with the *H0918* model, as it did not produce stable gaits with the other models. We train 5 reflex-based controllers with different initializations until convergence, while we use the most natural RL policy for each model and perform 20 roll-outs with randomized initial states to test the robustness. We chose this approach as reflex-based controllers are sensitive to the initial simulation state; different roll-outs would be almost identical if similar starting states were used.

While both approaches adequately match human kinematics, as quantified by the exp. match metric, with low energy consumption in the planar case, the reflex-based controller produces more natural gaits. However, when exposed to uneven terrain, the RL agent achieves an average distance of 10.42 m, which shows that it is much more robust than the reflex controller with an average distance of 2.46 m, see Table 1. Both controllers also induce similar average muscle activities over the gait cycle, with the RL agent inducing less smooth activity, shown in Figure 3.

With the same framework, we were also able to train agents to learn maximum speed running, by simply using the achieved velocity as the velocity reward in our reward function. Additionally, the action clipping and effort costs were omitted, as energy consumption is less critical for short maximum performance tasks. See Figure 2 for these results.

As a showcase of the extreme robustness of the RL agents, we generated a difficult suspension-bridge-terrain task with moveable environment elements that present dynamic perturbations, see Figure S3. We test the robustness of *H1622* and *H2190* RL controllers in this scenario, even though they were only ever trained on **flat** ground, and observe remarkable stability across the task. We report the data in Table 2 and in the videos.

**Table 2:** Maximum running velocity for different models and total achieved distance in the dynamic terrain

system	H0918	H1622	H2190	MyoLeg
max velocity	5.38	5.04	6.49	5.44
achieved distance	n.a.	9.87±4.27	10.45±4.77	n.a.

Velocities are expressed in ^m^/_s_, the terrain is related to Figure S3. We show the maximum speed over 20 roll-outs for each model. We do not examine this environment for the *H0918* model, as the 3D nature of the terrain is not applicable to it, and not for the *MyoLegV0* model, as the terrain was not implemented yet in the MuJoCo engine when the experiments were conducted.

**Figure 4:**
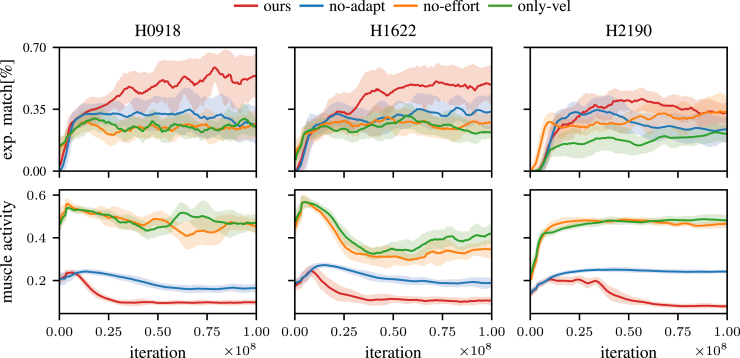
Cost function ablations We show several ablations of our cost function and plot the average match with experimental human data, as well as the average muscle activity. A natural gait is generally characterized by a large experimental match as well as minimal muscle activity. Different ablations are shown: The adaptive effort term is zero (α(t)=0): no-adapt. The entire effort cost term is zero $\left(c_{\text{effort}}=0\right)$ and we deactivate the action clipping: no-effort. We only reward with the velocity reward term ($c_{\text{effort}}=0$ & $c_{\text{pain}}=0$): only-vel. Only the combined cost function achieves a close resemblance to natural gait with low muscle activity. Leaving out the pain-related costs leads to the worst gait trajectories, while a combination of the effort cost terms and the adaptive cost term is needed to achieve the lowest muscle activity. All experiments report mean ± SD over 10 random seeds.

Note that we tried several alternatives to our approach which yielded worse results. We performed experiments with different reward terms such as a constant instead of an adaptive effort term, with metabolic energy costs^3^ or with a cost of transport^58^^,^^59^ reward. Even though these terms sometimes lead to small muscle activity during execution, the kinematics were further away from human data. We conjecture that energy minimization is not enough of an incentive for human-like gait if the learning algorithm is as flexible as an RL agent. See also Figure 4 for ablations of our reward function.

Larger effort term exponents, penalization of contacts between limbs or angle-based joint limit violation costs did not lead to better behavior. The prescription of hip movement at a certain frequency (step clock), keeping certain joint angles in pre-specified positions or minimizing torso rotation helped to achieve stable gaits, but prevented effort minimization and did not lead to natural kinematics.

### Ablations

In order to pinpoint the contributions of the different introduced mechanisms, we performed ablation experiments, see Figure 4. The considered variants include a reward function without the adaptive term (no-adapt: α(t)=0), a reward function without any effort terms (no-effort: $c_{\text{effort}}=0$), and a variant where all rewards except for the external task reward $r_{\text{vel}}$ were zero (only-vel). It can be observed that the exp. match metric is the highest with the inclusion of all reward and cost terms (ours). While the non-adaptive effort reward also leads to a smaller average muscle effort than the other reward functions, the final effort value is still larger. The initially high muscle effort for the ours-variant coincides with high muscular co-contraction levels, diminishing over time, similar to studies with human participants.^60^

## Discussion

The recently published DEP-RL^31^ approach was leveraged to learn feedback controllers for musculoskeletal systems. DEP-RL has been shown to achieve robust locomotion in several tasks, including running with a high-dimensional (120 muscles) bipedal ostrich model, by proposing an exploration scheme for overactuated systems. The learned behaviors, however, still exhibited unnatural artifacts, such as large co-contraction levels and excessive bending of several joints.

Here, we demonstrate that the combination of an effective RL algorithm^31^ with a reward function accounting for biologically plausible incentives results in gaits that closely resemble human walking. We achieve this by introducing a reward function that adapts its weights to the used model and is general enough to generate gaits across several models with up to 90 muscles in two and three dimensions and in simulators of differing biomechanical modeling accuracy **without** the need for manual tuning of the reward function for each model. Only the network size was decreased for the low-dimensional models to benefit from the computational speed up.

In our experiments with lower-dimensional 2D and 3D models (up to 22 muscles), the agents almost reach the naturalism of existing optimal control- and reflex-based frameworks.^4^^,^^5^^,^^28^ While the match to experimental human data is not perfect, it is closer than comparable methods in the literature that, similar to us, only use aggregated data to tune their reward function.^23^ In the complex 3D models with 80 and 90 muscles, which are substantially more challenging to control due to the large and redundant space of possible muscle excitations, our approach still achieved gaits with kinematics and GRFs similar to experimental human data, albeit with more artifacts. Achieving gaits in these complex models is a step toward applications in rehabilitation, neuroscience, and computer graphics requiring simulated human motor control with high-dimensional models in complex environments.

Striking is the robustness of the learned controllers exhibiting diverse stabilization strategies when faced with dynamic perturbations to an extent unseen in previous reflex-based controllers.^8^^,^^9^^,^^28^^,^^61^^,^^62^ As the used reward terms are considered plausible objectives for biological organisms, the general approach may also be applicable to different movements. Therefore, we see this study as a useful starting point for the community showing that RL in combination with biologically plausible objectives **is** a viable candidate to investigate the highly robust nature of complex human movements, even if no experimental data are available.

### Close-to-human gait with RL can be achieved with an adaptive energy cost

As the human biomechanical system is highly redundant, there are many possible solutions to walking at a defined speed. There exists strong evidence that natural human walking is in part driven by energy-efficiency^63^ and optimal control approaches have shown that natural walking kinematics can be achieved if energy optimality is considered in the cost function (Some also suggest that muscle fatigue could be the driving factor to explain the experimentally observed kinematic patterns^64^).

However, while model-predictive control and trajectory optimization methods provide valuable insight into singular trajectories and can deal with unexpected perturbations up to a certain degree, recent studies have shown that RL methods can generate more robust controllers^14^ and learn a large number of diverse movements.^20^

To extend the promising results in the field of robotics to high-dimensional biomechanical systems, while considering the minimization of muscular effort as a guiding principle, we introduce an adaptive reward function. A single reward term schedule adapts the weighting of the energy term in the reward function depending on the current performance, achieving energy-efficient gaits with more natural kinematics in a biomechanical setting. Moreover, the adaptation Algorithm (Algorithm 1) and all other reward terms and their weighting coefficients are general enough to work—without any changes—across 2D and 3D models with different numbers of muscles and even different levels of biomechanical modeling accuracy.

This is a significant step toward finding a general reward function and framework to generate natural and robust movements with RL in muscle-driven systems. Other RL frameworks that do achieve natural muscle utilization either consider low-dimensional systems^65^ or strongly rely on motion capture data^16^ to render the learning problem feasible. Our approach works **without** the use of motion capture data during training and with few and very general reward terms and therefore may generalize better to other movements.

In our opinion, there is only one comparable work.^23^ They achieved human-level kinematics on a planar human model with 18 muscles, by crafting a multi-stage learning curriculum affecting the weighting of seven reward terms. As this learning curriculum contains model-specific reward terms and adaptation procedures, we speculate that it would have to be hand-tuned for different models.

### The realism of learned behaviors is influenced by the embodiment of the agent

While our approach achieved higher robustness than reflex-based controllers and kinematics closer to natural walking than previous demonstration-free RL approaches, several discrepancies to natural walking remain, see Figure 2 and supplementary videos. The low-dimensional models (*H0918* and *H1622*) in general do not present proper ankle rolling, while the high-dimensional models (*H2190* and *MyoLegV0*) exhibit less passive leg-swing in the swing phase of the gait. We attribute these differences in behaviors to the physiological modeling differences between different models.

The behavior of the *MyoLegV0* model deviates stronger from human data than the *H2190*, although they are similar in terms of complexity. The *MyoLegV0* model lacks the double-peak structure in the GRFs and we also observed a tendency for unnatural lateral torso oscillations during walking and running, see Figure 3 and the videos. *MyoLegV0* uses a different muscle geometry from *H2190* and includes ellipsoidal collision objects for foot contact dynamics, which might increase learning difficulty. Alternatively, the more elaborate biomechanical features in Hyfydy, such as elastic tendons,^66^^,^^67^ non-linear foot-ground contact mechanics,^68^ variable pennation angles,^69^ or error-controlled integration, could account for the increased realism of the behaviors with the Hyfydy models.

Research on the contribution of biomechanical structures to the emergence of natural movement^70^^,^^71^^,^^72^^,^^73^ suggest that, in addition to the learning method and reward function, the biomechanical structures and modeling choices may play a crucial role in the accurate reproduction of human gait. This seems a plausible explanation for the increased realism in the Hyfydy models, as previous observations in predictive simulations suggest that e.g., an elastic tendon is beneficial for natural gait.^3^^,^^5^^,^^28^^,^^67^ We regard this as one interesting area of future research, which could help us better understand the fundamentals of the interaction between biomechanics and neuronal control in human locomotion.

### Conclusion

We achieved highly robust walking approaching human-like kinematics and ground reaction forces. While a better degree of accuracy was achieved in simpler models, we provide first promising results for difficult-to-control 80 and 90 muscle models that are of high interest for applications in rehabilitation, neuroscience, and computer graphics. Learning with the proposed reward function and RL framework allows for these results across several models of differing complexity and biomechanical modeling accuracy with only minimal changes in the hyperparameters of the method. We hope that this inspires researchers from both the biomechanics and the RL community to further improve on our approach and to develop tools to unravel the fundamentals of the generation of complex, robust, and energy-efficient human movement.

### Limitations of the study

We only performed experiments with two different behaviors, walking and running. It is unclear whether the reward function will generalize to other tasks. The learning problem did not include the control of arms or the incorporation of vision-based sensory inputs, both of which could make the tasks more difficult. Finally, while the overall match to experimental human data is quite close, methods optimized for a particular model might be able to achieve an even better fit. The influence of the distribution of male and female subjects in the used data was not investigated.

## Resource availability

### Lead contact

Further information and requests for resources and information should be directed to and will be fulfilled by Pierre Schumacher, (pierre.schumacher@cin.uni-tuebingen.de).

### Materials availability

This study did not generate new unique materials.

### Data and code availability

• This paper analyzes existing, publicly available data on human gait, accessible at https://simtk.org/projects/nmbl_running and https://github.com/tgeijten/scone-studio.
• The source code employed in the current research can be accessed on the GitHub page, and the link is given in the key resources table.
• Any additional information required to reanalyze the data reported in this paper is available from the lead contact upon request.

## Acknowledgments

Pierre Schumacher was supported by the International Max Planck Research School for Intelligent Systems (IMPRS-IS). This work was supported by the Cyber Valley Research Fund (CyVy-RF-2020-11 to D.H. and G.M.). We acknowledge support from the Open Access Publication Fund of the University of Tübingen.

## Author contributions

Conceptualization, P.S., G.M., D.F.B.H., and S.S.; methodology, P.S., G.M., D.F.B.H., T.G., V.K., and V.C.; software, P.S., T.G., V.K., and V.C.; writing – original draft, P.S., G.M., and D.F.B.H.; writing – review and editing, P.S., G.M., D.F.B.H., S.S., T.G., V.K., and V.C.

## Declaration of interests

V.K. and V.C. are senior research scientists at Meta AI. T.G. is the author and proprietor of the Hyfydy simulation software.

## STAR★Methods

### Key resources table

**Table:** 

REAGENT or RESOURCE	SOURCE	IDENTIFIER
**Deposited data**		

Human gait data.	Hamner et al.^57^	https://simtk.org/projects/nmbl_running

**Software and algorithms**		

Reinforcement learning algorithms	Schumacher et al.^31^ and the present study	https://github.com/martius-lab/depRL
Hyfydy models used in the present study	The present study	https://github.com/tgeijten/sconegym
SCONE.	The SCONE open-source software	https://scone.software/doku.php?id=start
MyoLeg model	MyoSuite	https://github.com/myohub/myosuite/

### Method details

#### Simulation engines

We use both the Hyfydy and the MuJoCo simulation engines for our experiments. Both engines differ in these key areas.

##### Musculotendon dynamics

The muscle model in Hyfydy is based on Millard et al.^74^ and includes tendon elasticity, muscle pennation, and muscle fiber damping. The MuJoCo muscle model is based on a simplified Hill-type model, parameterized to match existing OpenSim models^51^ and supports only rigid tendons and does not include variable pennation angles.

##### Contact forces

Hyfydy uses the Hunt-Crossley^75^ contact model with non-linear damping to generate contact forces, with a friction cone based on dynamic, static, and viscous friction coefficients.^76^ MuJoCo contacts are rigid, with a friction pyramid instead of a cone, and without separate coefficients for dynamic and viscous friction.

##### Contact geometry

The MuJoCo model uses a convex mesh for foot geometry, while in the Hyfydy models the foot geometry is approximated using three contact spheres.

##### Integration step size

Hyfydy uses an error-controlled integrator with variable step size, while MuJoCo uses a fixed step size and no error control. The average simulation step size in Hyfydy is around 0.00014s (7000 Hz) for the H2190 model, compared to the fixed MyoSuite step size of 0.001s (1000 Hz) for the *MyoLegV0* model.

#### State space

The state space, or sensory input, of the RL agents, are the Cartesian position and orientation of the pelvis, their velocities, internal joint angles and velocities, the center-of-mass velocity, the torso orientation, the pelvis height, the position of the feet relative to the pelvis and muscle fiber lengths, velocities as well as muscle force. We also zero the Cartesian pelvis position in the x-dimension to improve performance, similar to.^15^

#### Definition of reward terms

To facilitate the reproduction of our results, we give a detailed description and definition of all the used reward terms: The complete reward function is given by:

(Equation 5) $$r=r_{\text{vel}}-c_{\text{effort}}-c_{\text{pain}}\text{.}$$

The first term describes the task, in this case, it is a target velocity:

(Equation 6) $$r_{\text{vel}}=\left\{ \begin{array}{l} \exp\left[-{\left(v-v_{\text{target}}\right)}^{2}\right]\;\text{if}\;v<v_{\text{target}}\\ 1\;\text{otherwise}, \end{array}\right.$$

where v is the velocity of the pelvis in the forward direction.

The second term, responsible for minimizing the effort, is:

(Equation 7) $$c_{\text{effort}}=\alpha \left(t\right)\;\sum_{j=1}^{M}a_{m}^{3}+w_{1}\;\frac{1}{M}\sum_{m=1}^{M}{\left(u_{m}-u_{m}^{\text{prev}}\right)}^{2}+w_{2}\;\sum_{m=1}^{M}\left[a_{m}>0.15\right]\text{.}$$

where the sum is over all muscles in the respective model and [·] denotes the Iverson bracket, an indicator function that is 1 if the interior condition is true, and 0 otherwise. Note that the term encouraging smoothness of the excitations, leading with $w_{1}$, is averaged over all muscles, while the other terms are not. The reasoning is that the first term uses the adaptive term α(t), making additional weighting unnecessary, while the last term encourages sparse muscle activity. We want as few active muscles at all times as possible.

Lastly, the pain cost term is given by:

(Equation 8) $$c_{\text{pain}}=w_{3}\;\sum_{j=1}^{J}\tau_{i}^{\lim}+w_{4}\;\max\left(0,\frac{1}{W}\sum_{k=1}^{2}F_{k}^{\mathrm{GRF}}-1.2\right)\text{.}$$

The $w_{4}$-term sums the ground reaction forces between each foot and the ground and normalizes it by the total body weight W. Only values exceeding 1.2 are punished.

The $w_{3}$ term sums the forces with which joint limits are violated over all joints in the model. For the *MyoLegV0*, we did not consider internal DOFs of the patella for this cost, as we did not achieve good results when incorporating it. This is a natural cost term as joint limits in humans are not entirely stiff due to elastic tendons and other passive tissue.^45^ Both used simulation engines allow small violations of the joint limits which produce forces that drive the joint angle back to minimize the violations.

The experiments with the running task also contain a self-contact cost:

(Equation 9) $$c_{\text{contact}}=w_{5}\;\text{clip}\;(\sum_{\begin{array}{l} i\neq \text{ground}\\ j\neq \text{ground} \end{array}}\left|f_{c}\left(i,j\right)\right|,0,100)/100\text{,}$$

which adds all contact forces between bodies i and j except for collisions with the ground. We consider this cost to belong to the pain cost term, as strong and repetitive collisions between limbs during high-performance running would lead to injury in a human. We clip and scale the contact forces to the range $c_{\text{contact}}\in \left[0,w_{5}\right]$. Unscaled contact forces can vary wildly for different simulation engines and might interfere with training stability. In this way, only strong and potentially painful self-contacts are considered, while weaker collisions can be safely ignored by the learner.

#### Training curves

Here we present more detailed results about the training evolution in Figure S1. We plot the experimental match percentage between the collected gait-cycle averaged data and experimental human data, the muscle-averaged effort, the training returns, and the weight that the effort-reward term has over training. This weight is adapted over time and depends on the agent’s performance. It increases slower for the complex models and saturates at smaller values. It can also be seen that the returns for the *MyoLegV0* are generally smaller than for the other models. We observed that there was more variance over training and over different seeds for the *MyoLegV0*-agent, leading to much smaller averaged returns. It was still possible to find a training checkpoint that achieved robust, close-to-human-like walking for this model. Here we present more detailed results about the training evolution in Figure S1. We plot the experimental match percentage between the collected gait-cycle averaged data and experimental human data, the muscle-averaged effort, the training returns, and the weight that the effort-reward term has over training. This weight is adapted over time and depends on the agent’s performance. It increases slower for the complex models and saturates at smaller values. It can also be seen that the returns for the *MyoLegV0* are generally smaller than for the other models. We observed that there was more variance over training and over different seeds for the *MyoLegV0*-agent, leading to much smaller averaged returns. It was still possible to find a training checkpoint that achieved robust, close-to-human-like walking for this model.

#### Effort cost adaption

The effort cost adaptation considers the current external task performance as a criterion for adaption. This means that only the $r_{\mathrm{vel}}$ term in Equation 1 is used to change the effort reward weight. We provide details for this mechanism in Algorithm 1.

Given a performance threshold θ, a smoothing parameter β, a change in adaptation rate Δα and a decay term λ, we initialize a running mean for the episode return $r_{\text{mean}}$, the switching indicator $s_{\text{mean}}$ and set the initial adaptation rate to $a_{t}$ equals zero. The effort weight $a_{t}$ increases by Δα if the running mean of the episode return $r_{\text{mean}}$ is above the defined threshold θ and the switch indicator $s_{\text{mean}}$ is above 0.5. The indicator $s_{\text{mean}}$ slowly moves towards 1 if the average episode return is above the threshold and slowly moves towards 0 if it is not. It therefore indicates whether the current episode performance has only been achieved for a short time or if the agent has been consistently well performing. If the performance is above the threshold, but only recently so, as indicated by $s_{\text{mean}}$, we decrease the change in adaptation rate Δα by multiplying it with the decay term λ. In the case of consistently bad performance, the effort cost decreases.

This mechanism increases the effort weight as long as good task performance can be achieved and decreases if the agent does not achieve it reliably anymore. The rate decay was introduced to prevent performance oscillation without a decrease in the used effort. We observe that the agent only learns low-effort behaviors if the effort weight has been high for many training iterations, while the absolute value of the weight as well as the adaptation speed depends on the used model and the task. Our mechanism is therefore capable of creating a personalized learning schedule for each of the evaluated biomechanical models.

We have observed that for the models with a small number of DOFs, the effort weight goes up rapidly and the rate of increase will only get adjusted once or twice for an entire training run. For the high DOF models, and more difficult tasks which require longer training time, the learning progress will collapse many times until the rate of increase of the effort weight has slowed down significantly. In this manner, we benefit from a fast effort adjustment when network training is fast and the task is easy, while difficult training runs automatically adjust to a slow increase of the effort weight over time. See also Figure S2.

#### Running

We performed maximum-speed running experiments with every model. While most reward terms remained identical to the natural walking case, we replace the external task reward by the velocity of the center of mass $r_{\text{vel}}=v$ and removed energetic constraints such as the muscle excitation clipping and the effort cost term. The gait-cycle- and leg-averaged kinematics are shown in Figure 2. As this task is a maximum performance movement, we have equalized the forces between the Hyfydy- and MuJoCo-based models, as the Hyfydy-models in the main experiments are generally based on experimental data with weaker maximum isometric muscle forces.^46^ Note that we added a negative reward for self-collision forces for the running tasks, as the agents would often cross their legs and hit them against each other, thereby hopping instead of running. The self-collision forces are clipped between f∈[−100,100] and normalized by dividing by F=100. We use a reward coefficient of $\omega_{\text{contact}}=-10$ for all environments.

Even though there remains a stronger discrepancy between the produced kinematics and the experimental data than for walking, the hip movement and GRFs are generally well aligned for the Hyfydy-models. The *MyoLegV0*-model presents very strong lateral torso oscillations during running, see also Figure 3. In future work, biological objectives such as head stabilization or the inclusion of arms in the model might minimize some of these artifacts. See Table 2 for the maximum running velocities for each model.

We also performed robustness experiments on a challenging obstacle course, see Figure S3 and supplementary videos.

#### Hyperparameters

Used hyperparameter settings for the RL agent, DEP and the cost function are shown in Table S1. Non-reported RL values are left to their default setting in TonicRL.^77^ See^31^ for an explanation of the DEP-specific terms. The RL parameters were held constant, except for an increase in network capacity for *H2190* and *MyoLegV0*.

#### Effect of network size

We observed that the models with a large number of DOFs generally require larger networks to properly train. Although the less complex models could potentially be trained with larger network sizes and a good performance would be reached eventually, this increases training time tremendously. We therefore choose the smallest network size that can reach a good performance for each model, to provide a quick-to-train baseline for other researchers to build on. Performing more difficult tasks with the less complex models may also require the use of bigger network sizes.

### Quantification and statistical analysis

Basic statistical analysis was performed using numpy (version 1.26.4).^78^ Mean and standard deviation are indicated whenever an average over several rollouts or policies was performed. Additional information not contained in this section can be found in the figure legends.

#### RL training and evaluation

All RL policies are trained with the DEP-RL (https://github.com/martius-lab/depRL) software framework, reliant on the TonicRL (https://github.com/fabiopardo/tonic) implementation. We train each policy with 10 random seeds, for which the performance is plotted as averages with the standard deviation in shaded areas for all training performance plots. The controllers train for 100 million environment interactions each. The used reflex-controllers are included in the SCONE (https://scone.software/doku.php?id=ref:reflex_controller) software package.

For the evaluation of the most human-like gait, we take all training checkpoints after training and compute the experimental match metric by creating 5 rollouts of a 10 s walk for each checkpoint. The seed and training checkpoint with the largest value are taken for the evaluation.

For the robustness experiments, we randomly generate a terrain with 10 tiles of 1 m length and random slopes between ±5deg. This terrain stays otherwise fixed for all evaluations.

To test the robustness of the reflex controller and the RL agent, we optimize 5 controllers with different initializations until convergence, while we use the most natural RL policy for each model and perform 20 roll-outs with randomized initial states of the agent. The walked distance serves as a robustness metric.

#### Data preparation

The used datasets contain motion capture and ground reaction force (GRF) recordings of adult males walking and running on a treadmill at 1.2 m/s and 5.2 m/s respectively. All the data are available through public repositories (https://simtk.org/projects/nmbl_running) or are freely available with the SCONE software (https://scone.software/doku.php?id=start). We split all the trajectories into gait cycles by measuring the time from ground contact to ground contact of the left or the right foot via spikes in the respective GRFs. We consider a foot to be in contact with the ground if the computed normal force is larger than a threshold $f_{\text{thr}}=0.001$. The same procedure is repeated for the gaits produced by the RL agent.

We then take the maximum and the minimum joint angles or GRF values of all the gait cycles of all the participants moving at a certain speed and check whether the values produced by the RL agent fall inside the range of the human experimental data. The percentage of the gait cycle for which the produced values are inside the range is finally used as a performance metric for optimization. This procedure is similar to the method included in the SCONE repository (https://github.com/opensim-org/SCONE/blob/6c8e8e3e45615ae2065a27243cfd8fbb778195a2/src/sconelib/scone/measures/GaitMeasure.cpp\#L71). We use the joint angles of the hip, knee, and ankles as well as the GRFs for our optimization. Values from the left and the right leg are averaged. The metric thereby implicitly values symmetric gaits higher than asymmetric gaits, for which the values do not align as well.

While this approach uses experimental data to guide the hyperparameter selection, no recordings from human subjects are used during the learning process.

## Supplemental information

Document S1. Figures S1–S3 and Table S1

Video S1. Walking and running performance for different models and environments, related to Figure 2
