# Supplementary material for: Emergence of natural and robust bipedal walking by learning from biologically plausible objectives
Source: iScience. 2025 Mar 11;28(4):112203. doi: 10.1016/j.isci.2025.112203 (PMC12002607; doi:10.1016/j.isci.2025.112203)
Supplement: Document S1. Figures S1–S3 and Table S1 [file mmc1.pdf]

iScience, Volume *28*

## Supplemental information

# Emergence of natural and robust bipedal walking by learning from biologically plausible objectives

**Pierre Schumacher, Thomas Geijtenbeek, Vittorio Caggiano, Vikash Kumar, Syn Schmitt, Georg Martius, and Daniel F.B. Haeufle**

# Supplemental information

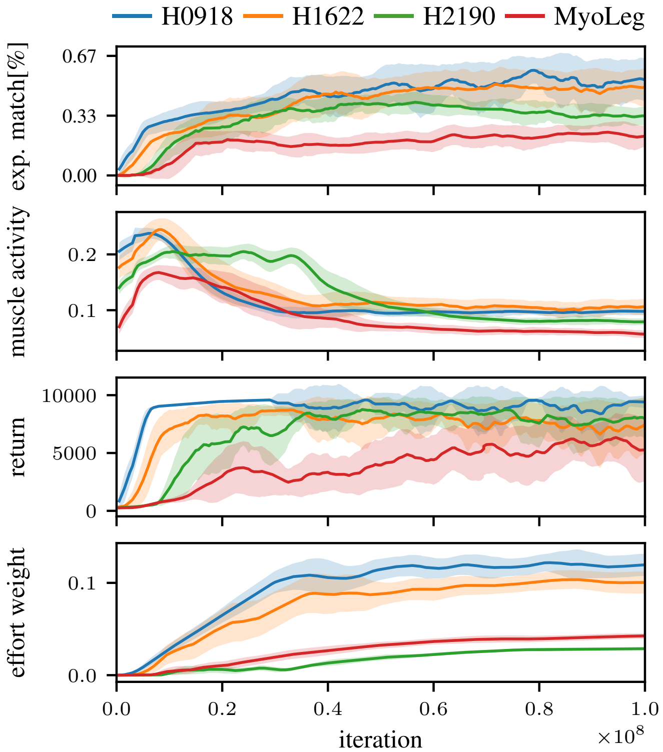


Fig. S1: **Training curves for the walking task.** We present the evolution of the match with experimental data (exp. match), the task performance, the averaged muscle activity, and the effort cost weight. The cost weight increases more slowly for the more complex models, showing its adaptive nature. All experiments report mean ± SD over 10 random seeds.

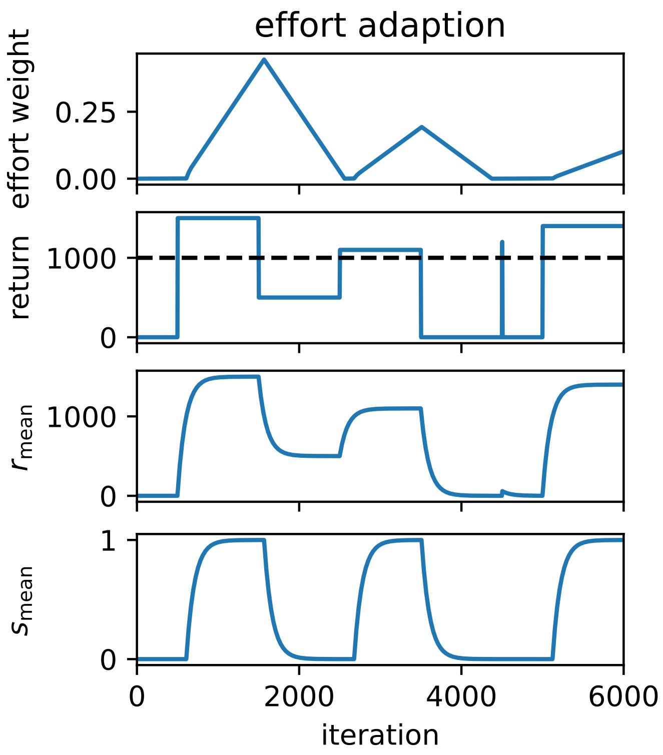


Fig. S2: **Adaptive effort weight adjustment.** The adaption mechanism causes a decrease in the effort cost learning rate when the task performance oscillates. We show an illustrative example with $\beta = \alpha = 0.99$, $\Delta\alpha = 9 \times 10^{-4}$ and $\theta = 1000$. The dashed line marks the threshold.

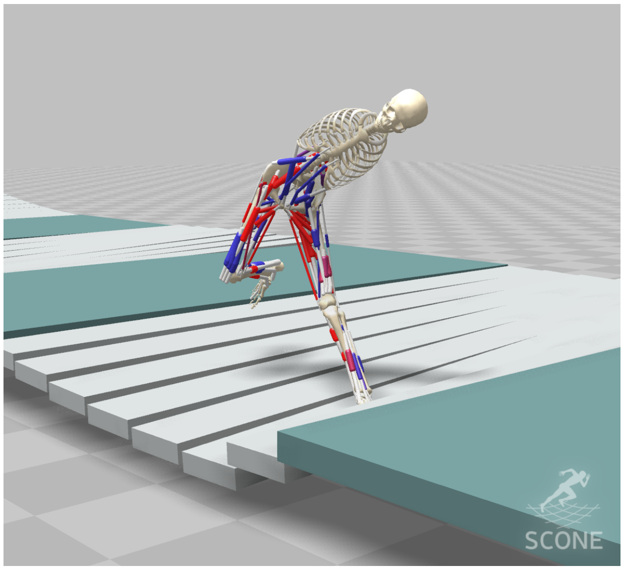


Fig. S3: **Dynamic terrain for running.** We probe the robustness of our policies trained for the *H1622* and the *H2190* with challenging obstacles. The tiles of the bridge rotate around the central axis and hang downwards, similar to a suspension bridge. The agents were trained on **flat** ground and only have access to proprioceptive feedback.

TABLE S1: Hyperparameters for all algorithms, related to Fig. 1.

| **DEP Settings** | |
|---|---|
| **Parameter** | **Value** |
| $\kappa$ | 1200 |
| $\tau$ | 40 |
| Buffer size | 200 |
| Bias rate | 0.002 |
| $s4avg$ | 2 |
| Time dist ($\Delta t$) | 5 |
| $p_{switch}$ | $3.71 \times 10^{-4}$ |
| $H_{DEP}$ | 8 |
| Test episode | 3 |
| Force scale | n.a. |

| **MPO Settings** | |
|---|---|
| **Parameter** | **Value** |
| Buffer size | $1 \times 10^{6}$ |
| Batch size | 256 |
| Steps before batches | $2 \times 10^{5}$ |
| Steps between batches | 1000 |
| Number of batches | 30 |
| $n$-step return | 1 |
| $n$ parallel | 20 |
| $n$ sequential | 10 |
| Hidden layers | 2 |
| Hidden sizes | 256 |
| $l_{actor}$ | $3 \times 10^{-4}$ |
| $l_{critic}$ | $3 \times 10^{-4}$ |
| $l_{dual}$ | $1 \times 10^{-2}$ |

| **MPO Settings Changes for H2190 and MyoLegV0** | |
|---|---|
| **Parameter** | **Value** |
| Hidden sizes | 1024 |
| $l_{actor}$ | $3.53 \times 10^{-5}$ |
| $l_{critic}$ | $6.08 \times 10^{-5}$ |
| $l_{dual}$ | $2.13 \times 10^{-3}$ |

| **Cost Function Settings** | | |
|---|---|---|
| **Parameter** | **Value** | **Meaning** |
| $\omega_1$ | 0.097 | Action smoothing |
| $\omega_2$ | 1.579 | Number of active muscles above 15% activity |
| $\omega_3$ | 0.131 | Joint limit torque |
| $\omega_4$ | 0.073 | GRFs above 1.2 BW |
| $\omega_5$ | 10 | Self-contact (only running) |
| $\Delta\alpha$ | $9 \times 10^{-4}$ | Change in adaptation rate |
| $\theta$ | 1000 | Performance threshold |
| $\beta$ | 0.8 | Running avg. smoothing |
| $\lambda$ | 0.9 | Decay term |
